# Modeling of time dependent localized flow shear stress and its impact on cellular growth within additive manufactured titanium implants

**DOI:** 10.1002/jbm.b.33146

**Published:** 2014-03-25

**Authors:** Ziyu Zhang, Lang Yuan, Peter D Lee, Eric Jones, Julian R Jones

**Affiliations:** 1 Department of Materials, Imperial College LondonLondon, SW7 2AZ, UK; 2 Manchester X-ray Imaging Facility, School of Materials, The University of ManchesterManchester, M13 9PL, UK; 3 Department of Engineering, University of LiverpoolLiverpool, L69 3GH, UK

**Keywords:** fluid shear stress, cellular growth, numerical modeling, titanium porous structures, additive manufacturing

## Abstract

Bone augmentation implants are porous to allow cellular growth, bone formation and fixation. However, the design of the pores is currently based on simple empirical rules, such as minimum pore and interconnects sizes. We present a three-dimensional (3D) transient model of cellular growth based on the Navier**–**Stokes equations that simulates the body fluid flow and stimulation of bone precursor cellular growth, attachment**,** and proliferation as a function of local flow shear stress. The model's effectiveness is demonstrated for two additive manufactured (AM) titanium scaffold architectures. The results demonstrate that there is a complex interaction of flow rate and strut architecture, resulting in partially randomi**z**ed structures having a preferential impact on stimulating cell migration in 3D porous structures for higher flow rates. This novel result demonstrates the potential new insights that can be gained via the modeling tool developed, and how the model can be used to perform what-if simulations to design AM structures to specific functional requirements.

## INTRODUCTION

Implants for bone augmentation are expected to stimulate bone ingrowth to enable fixation to the host bone, and their porous surfaces facilitate osteogenic cell recruitment to improve such fixation.^1^ Both the mechanical environment and interstitial fluid flow can significantly affect bone tissue remodeling.^2^–^5^ Vascularization and osteogenic cell proliferation plays a key role in osteogenesis^6^ and is promoted in the bone healing process by interstitial fluid flow in periosteum and surrounding tissues.^7^ Johnson et al.^8^ hypothesized that fluid flow-induced shear in bone regulates continuous and rapid release of nitric oxide from osteoblasts and the vascularization introduced by the fluid flow may stimulate bone formation. Meanwhile, bone deformation caused by the mechanical loading also produces interstitial fluid flow in the bone tissue.^9^,^10^ Weinbaum et al.^9^ suggested that osteocytes can be stimulated by the mechanical induced fluid shear stress and thus promoting osteoblast migration and proliferation.

**FIGURE 1:**
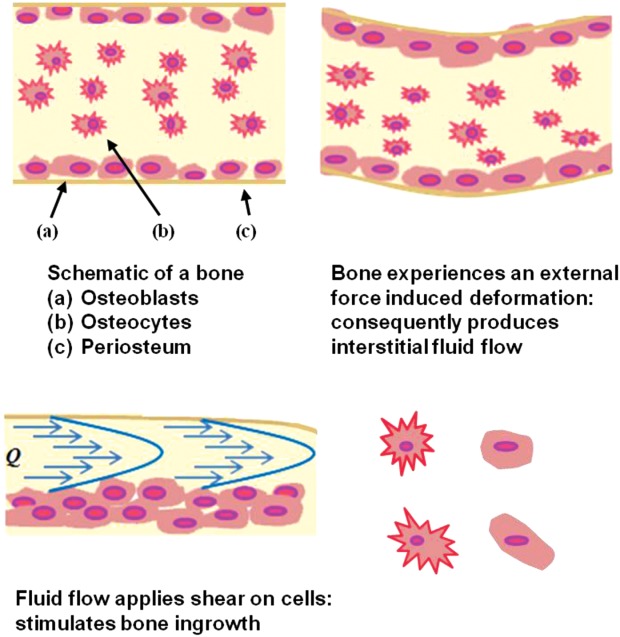
Schematic of bone and mechanical deformation induced interstitial fluid flow. Fluid-induced shear stress comes from the mechanical movement, upregulating cell proliferation/attachment and hence the cellular growth (after Carvalho et al.^48^). [Color figure can be viewed in the online issue, which is available at http://wileyonlinelibrary.com.]

Figure 1 shows a schematic of bone with mechanical deformation induced interstitial fluid flow. The mechanical movement induces the interstitial fluid flow and applies shear on cells. Similar to the mechanical loading system, flow-induced shear stress applied on the implant structure via *in vitro* three-dimensional (3D) perfusion systems has also been found to have important stimulatory effects on cell and tissue growth.^3^,^11^,^12^ Raimondi et al.^13^,^14^ was first to perfuse the culture medium through the 3D inner structure of the chondrocyte-seeded scaffold and predicted that a wall shear stress in the range 1.5–13.5 mPa was required for the stimulation of higher cell viability. By assessing the MC3T3-E1 osteoblast-like cell viability qualitatively by confocal microscopy and measuring the DNA content within the scaffolds, Cartmell et al.^15^ suggested that for a positive effect on seeded cell viability and proliferation *in vitro*, fluid shear stress ranging from 0.05 to 25 mPa was desired. Knowledge of how shear stress relates to bone precursor cell migration, attachment and proliferation (termed *cellular growth* from here on) in various design architectures can help optimize both implant design and manufacture. An understanding of fluid-induced shear stress within porous structures and induced bone ingrowth, is therefore of great importance and has been studied numerically by several prior authors.^14^,^16^–^23^

Raimondi et al.^13^ developed a 2D computational fluid dynamics (CFD) model to characterize the macroscopic flow through a scaffold made of hyaluronic fibers. The model was a first attempt to provide the correlation between the fluid shear stress and cultured cell response. It predicted a median shear value of 3 mPa at a 44.2 µm/s flat inlet velocity, however, the accuracy of the model was limited by the 2D domain.

Botchwey et al.^24^ used Darcy's law to estimate the shear stress analytically in a 3D porous scaffold, and Coletti et al.^20^ calculated the macroscopic average shear stress by modeling the flow in a homogeneous scaffold volume. With the development of microtomography (µCT) being applied to tissue engineering scaffold imaging and quantification,^25^,^26^,^50^ the simulation of flow in 3D constructs with real pore architectures improved dramatically.^16^,^27^,^28^ Raimondi et al.^16^ calculated the shear stress acting on the outer surface of the internal pores, using a CFD model based on a partial volume from µCT images, to be in the range 4.6–56 mPa. These results suggested a strong correlation between the hydrodynamic shear and the invoked biosynthetic response in chondrocyte systems. Porter *et al*.^19^ used 3D lattice Boltzmann simulations to investigate the flow in cylindrical scaffolds as a function of flow rates, and they found an average shear stress of 0.05 mPa was required to have stimulating effect on cell proliferation, and that higher shear stress would lead to subsequent upregulation of osteoblast growth.

Cioffi et al.^18^ developed a CFD model to evaluate the shear stress acting on scaffold walls based on higher resolution µCT images of a polyester urethane scaffold. Various flow rates were simulated in this model and the calculated shear stresses varied between 0 and 40 mPa over the scaffold surfaces. The same group then developed a combined macroscale/microstructured model to investigate the effect of the flow rates and scaffold microstructures on shear stress and oxygen consumption rates in the central region of the scaffold.^29^ Their model suggested that a flow rate of 0.3 mL/min, at which 95% of the scaffold surface area experienced shear stresses less than 6.3 mPa, would maintain the oxygen supply above the anoxic level. While µCT has allowed significant improvements in capturing geometric effects on flow, these have yet to be coupled with time dependent simulation of the influence of cell parameters, such as migration and attachment rate, matrix deposition, and the resultant time dependent reduction in flow channel directions. All these issues are crucial to the correlation of fluid induced shear stress and osteoblast growth.

Chung et al.^22^ developed a mathematical model which incorporated nutrient transport to predict the macroscopic average shear stress with a fivefold increase in correlation to cell growth. Liu et al.^21^ presented a mathematical model which applied the Brinkman equation to describe the local fluid flow. Their work incorporated the nutrient transfer and the flow rates with cellular growth. Recently, Lesman et al.^23^ considered the time dependent proliferation and matrix production from preseeded fibroblasts inside a porous structure by adding cell-layers of constant thickness onto the pore periphery, which however in reality, greatly depends on local shear stress acting on the cells. All of the prior studies focused on macroscopic shear stress predictions; however, there has not been a comprehensive study on the local shear stress effect on cellular growth which inter-relates time dependent flow simulation with flow-induced shear stress distribution on a microscopic level.

Quantifying local flow shear stress to predict cellular growth at microscopic level, and then feeding the results growth back into flow simulations offers a better understanding of the complex coupling of how flow and porous scaffold strut architecture impact osteogenesis. In this study, a 3D microscale numerical model has been developed that simulates the fluid flow induced shear stress, time dependent cellular growth and the inter-relationship between the two dynamic factors. The model was then applied to study the influence of strut architecture design in AM Ti implants, with the inter-relationship between the flow-induced shear stress and the time dependent cellular growth at the microscopic level being determined in different implant structural designs.

## MATERIALS AND METHODS

A microscale model was developed to study the flow induced shear stress and cellular growth by solving the Navier–Stokes equations interactively with the cellular growth. The CFD model employed here was based on a prior 3D open-source microscale three-phase flow model, µMatIC, which incorporated momentum and mass transport in liquid–solid–gas phases.^30^,^31^ Therefore, only a summary of how the mass transport was simulated in the model is given below (for details see Refs.^31^–^36^) with details of the development of fluid shear stress evaluation and henceforth cellular growth prediction.

### Momentum and mass transport

We assumed the laminar flow occurring through porous media materials and the fluid is incompressible and Newtonian. Based on the control volume fixed in space, the momentum equation takes the form:^37^
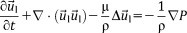
1where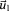 is the velocity vector in fluid, *t* is the time step, *P* is the pressure, ρ is the fluid media density, and *µ* is the fluid media viscosity. In order to unify the equation in the entire domain including fluid, bone and growing cells, the velocity field was defined as
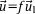
2where *f* is the fraction of fluid in a single control volume. When *f* = 1, the volume represents pure fluid in the porous space; when *f* = 0, it represents the bone structure itself, which ensures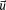 = 0 in the bone; and when 0 < *f* < 1, it represents the growing cell at the surface. Therefore, this transition from fully liquid (*f* = 1) to fully dense bone (*f* = 0) can represent the attachment of cells and their subsequent densification.

The conservation of mass equation, therefore, applied:
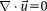
3

The flow governing equations above were solved by a projection method^38^ based on the regular orthogonal grid mesh using a control volume method. The Poisson equation for pressure, as deduced using the projection method, was solved using the preconditioned conjugate gradient solver. In order to take into account the momentum sink at the bone/fluid interface, the standard projection method was modified in the following way. The intermediate velocity, u*, explicitly evaluated from the previous time step and the pressure gradient ∇*P* were multiplied by the liquid fraction to obtain the new velocity,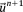:
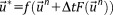
4
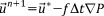
5where *F* is the discrete convection and diffusion term in the Navier–Stokes equations. The method has been validated against analytical solutions.^39^ This modification does not introduce extra computational effort but provides a dynamic solution to the transient cellular growth.

### Shear stress and cellular growth model

Shear stress on the bone surface was calculated through the velocity gradients neighboring the growing cells. Velocity components were specified at the center of each cell, and gradients were evaluated linearly along velocity nodes to the center. Based on the definition of fluid shear being the components of stress coplanar with the cross section of a control volume,^40^ we defined the shear stress in the *x* direction as:
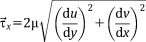
6where *u* and *v* are the velocity tensors on the tangential plane of the fluid flow. A similar definition is then applied to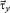 and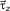.

The local shear stress magnitude was calculated as
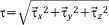
7

For a simpler calculation and less intensive computational requirement, we set the subsequent cell growth rule as a simplified linear relationship with the shear stress (original reference of the concept in Ref.^21^). The absolute growth (%) on each grid was defined as:
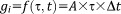
8where *A* is a constant, termed the growth factor;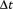 is the time step. The value of the growth factor was chosen so that the maximum growth rate matches previous recorded values in literature.^22^,^41^

**FIGURE 2:**
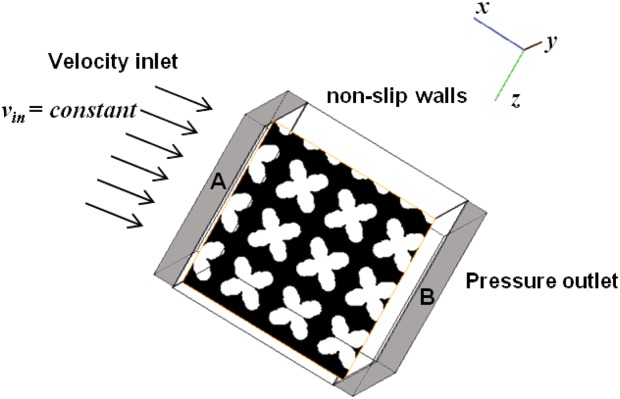
Schematic of the flow system used for the cellular growth simulation with boundary and initial conditions (regions A and B: fluid buffer zones). [Color figure can be viewed in the online issue, which is available at http://wileyonlinelibrary.com.]

Figure 2 shows the schematic of the boundary and initial conditions used in the simulations. A fully liquid region was placed up- and downstream to act as a fluid buffering zone and to allow the flow to stablize on the upstream and downstream flow faces. Up/downstream end faces of the buffer zone in the desired flow direction were set as fluid inlet/outlet boundaries. Four different constant inflow velocities, 0.02, 0.05, 0.1, and 0.2 mm/s were simulated (note all producing laminar, low Reynolds number flows). A pressure outlet was imposed on the outlet boundary. A no-slip condition was used on the predefined bone–fluid interface and zero-flux on other bounderies. The other simulations parameters are given in Table 1.

**TABLE 1:** Parameter Values Used in the Model

Property	Value	Unit	References
Initial inflow velocity (*v*_in_)	2 × 10^−5^	m/s	
	5 × 10^−5^		
	1 × 10^−4^		
	2 × 10^−4^		
Medium density (ρ)	1009	kg/m^3^	Coletti et al.^20^
Medium viscosity (*µ*)	8.4 × 10^−4^	kg/ms	Coletti et al.^20^
Growth factor (*A*)	1.5 × 10^−4^	–	
Max growth rate	1.5 × 10^−5^	s^−1^	Coletti et al.^20^
Min (critical) shear stress (τ)	0.05	mPa	Cartmell et al.^15^
Max shear stress (τ)	56	mPa	Raimondi et al.^16^
Grid size	36	µm	

It should also be noted that, as previously reported by Cartmell et al.^15^ and Raimondi et al.,^16^ once the shear stress becomes greater than 0.05 mPa, cell proliferation/stimulation will be promoted. However, if the shear stress becomes too large, exceeding 56 mPa, cells tend to be washed out and therefore it slows down the cellular growth rate. Therefore, a minimum and a maximum shear stress constraint were incorporated in the model. In this case, the growth vanishes when the calculated shear stress magnitude lies outside the range (shear stress <0.05 mPa or >56 mPa). The simulation was run both with and without the upper constraint of 56 mPa so that the effect of high shear stress on decreasing the cell growth can be quantified.

### Implant microstructures

In this study, we compared two different porous Ti implant structures designed by Mullen et al.,^42^ with regular and randomized strut ordering respectively, as a demonstration of the applicability of the simulations to help analyze real designs. The implants were constructed using the unit cell (UC) approach.^43^ A brief description of the design procedure is given below, for full details see Mullen et al.^42^,^43^ Note that the variability has been quantified both by Mullen et al. and also in terms of baseline flow properties in Zhang et al.,^44^ therefore only one example of each design type will be studied, and only as a virtual model.

All porous structures were designed based on a computer-aided design (CAD) model by using the Manipulator© software suite (University of Liverpool, UK). The 3D structure was first completely filled with cubic UCs of unit side of 600 µm. Then all UCs were filled with a connected lattice structure by joining sets of vertices and vectors in 3D space to form regular octahedrons (allows tessellation in 3D). Further on, the Cartesian coordinates of the lattice points were perturbed by ±30% in the *x*, *y*, and *z* directions, whilst maintaining connectivity, to produce a 30% randomized structure (Note that the randomized structures are actually pseudo-random because the randomness is fully reproducible.) When producing actual components, the selective laser melting (SLM) additive manufacturing process is used. During SLM, the vertices and vectors were sliced at 50 µm intervals to locate laser melting points on all layers. To produce virtual parts, we simulated this process by voxelizing the SLM input file containing the coordinates of the laser melting nodes producing a 3D model implant. Using an inhouse convolving code,^49^ the melting nodes were first located as foreground voxels and a perfect sphere kernel was then chosen to perform convolution on these nodes throughout the volume to form the struts phase. All the other voxels are assigned as background voxels indicating the void phase. The grid mesh is therefore based on the voxelized CAD volume.

Both regular and 30% randomized structures form completely open porosity that is fully connected to the surface. The overall porosities of the two test samples were kept similar as 67.5 and 69.2%, respectively. The effects of randomising the regular lattice are that the mode value of pore size increases from 203 to 302 µm and the overall distribution is shifted to a wider range. This has brought about a difference of 3.4% in their intrinsic permeability property.

## RESULTS

The model was applied to calculate the cellular growth in the regular and 30% randomized structures with similar porosities characterized based on the 3D CAD design. For each sample, we calculated the velocity profiles, the shear stress magnitude and distribution, the pressure drop, the growth rate and corresponding volume fraction of cellular growth over 120 h at each inflow velocity.

**FIGURE 3:**
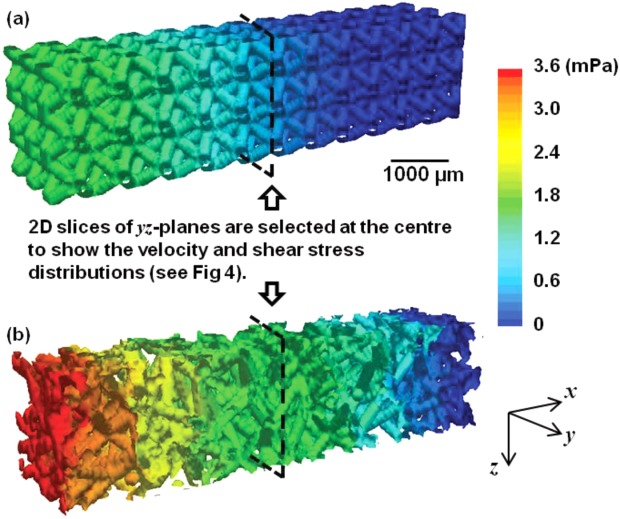
3D volume images showing the overall pressure changes in the regular and the randomized structures (inflow velocity: 0.02 mm/s). [Color figure can be viewed in the online issue, which is available at http://wileyonlinelibrary.com.]

The overall pressure drop in both implant structures at inflow velocity of 0.02 mm/s are shown in Figure 3. Pressure in the 3D volume of the regular design (0% randomization) varies from 0 to 1.5 mPa. Simulation in the 30% randomized structure reveals a maximum pressure variation 2.4 times greater than the regular due to the uneven distribution of velocities of fluid passing through more torturous channels.

**FIGURE 4:**
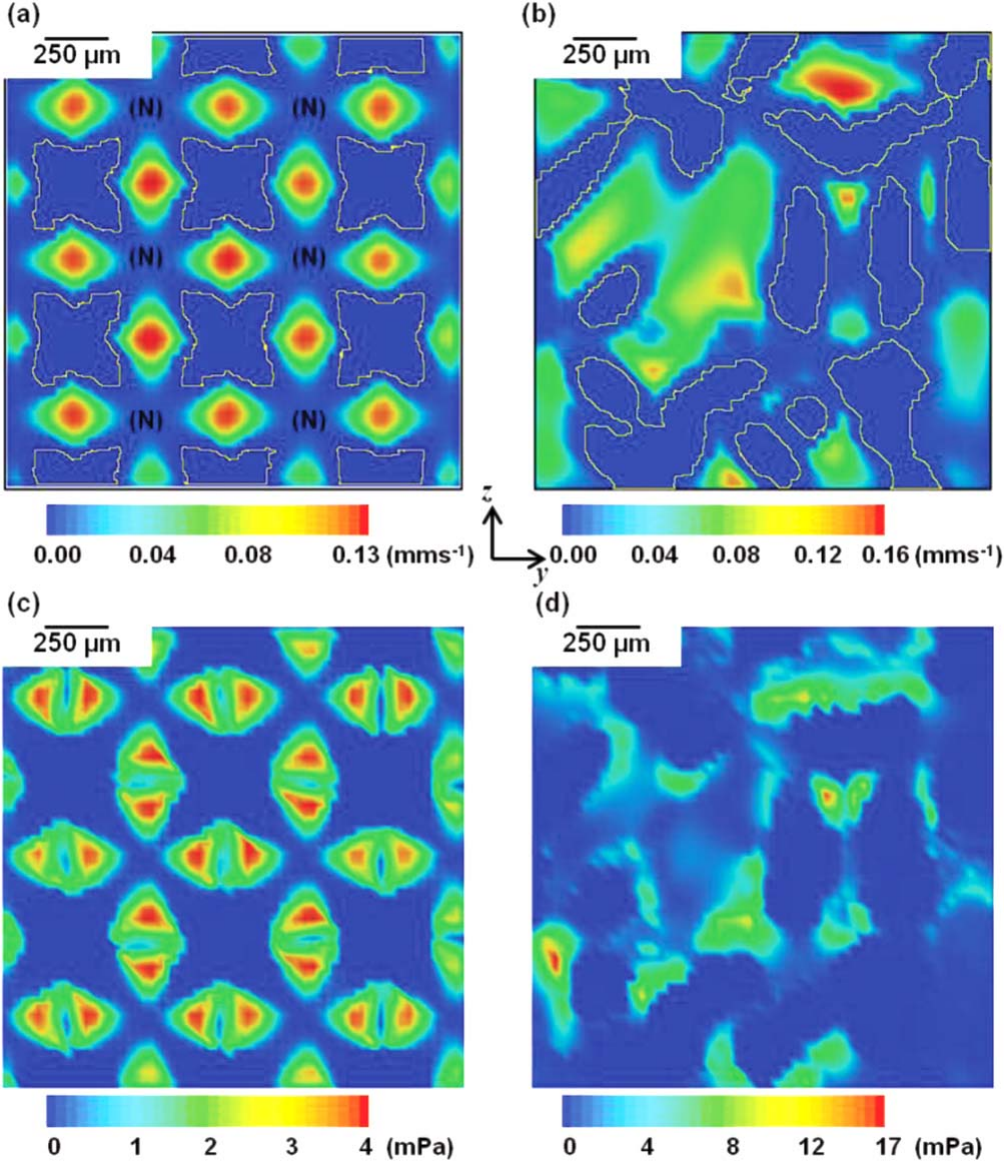
(a,b) 2D cross-sectional views of local velocity distributions in the regular and 30% randomized structures. (c,d) 2D cross-sectional views of local shear distributions in the two structures (inflow velocity: 0.02 mm/s). [Color figure can be viewed in the online issue, which is available at http://wileyonlinelibrary.com.]

Figure 4 shows selected 2D cross sectional views of the velocity and shear stress distributions from central slice of the 3D model for both the (a) regular and (b) 30% randomized structures when the inflow velocity is 0.02 mm/s. In the regularly ordered implant structure, higher velocity flow occurs in the narrow channels with weak flow in the open channel [labeled as region ‘(N)' in Figure 4(a)]. The shear stress is almost zero in ‘(N)' regions. The maximum values of velocity along the flow direction within the regular (0.13 mm/s) and randomized structures (0.16 mm/s) exhibit a difference of 23.1%. The average velocity throughout the entire structure is also compared: the 30% randomized structure has a higher average velocity value of 22.7% greater than that of the regular structure at the final stage of growth. Higher shear stress values are seen at the locations where the changing in velocity is significant. The maximum value of shear applied on the solid in the randomized structure is 3.4 times greater than the regular. Note that we ran a CFD simulation in Fluent on same structures without cells to validate the initial stage results, and the overall pressure changes and velocities agreed with each other within experimental error.

**FIGURE 5:**
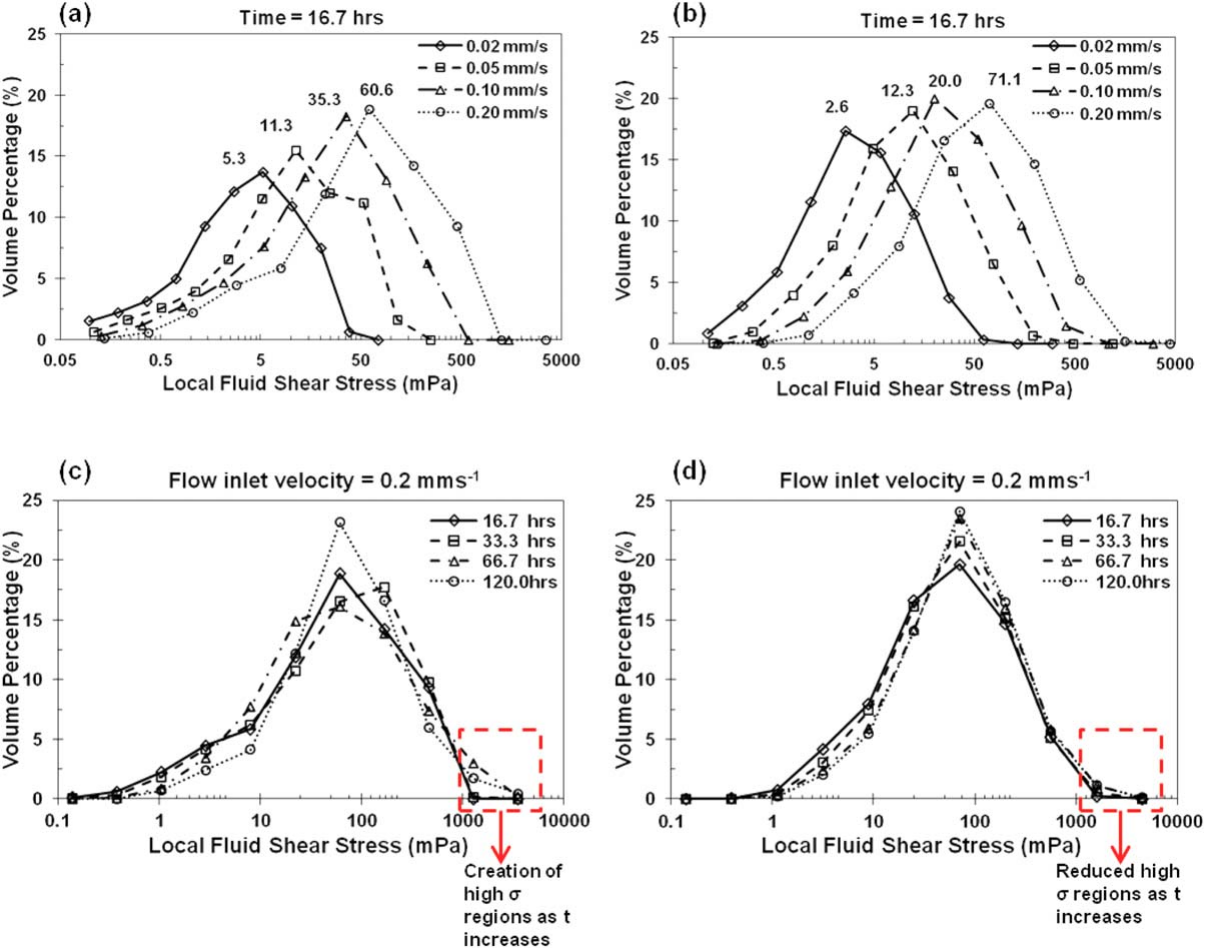
Local shear stress histogram distributions at different time points with the vertical axis showing the frequency density within a given shear range (log-based bin range). (a,b) At time point 16.7 h, shear distributions at four different inflow velocities in the regular (a) and 30% randomized (b) structures. (c,d) At inflow velocity 0.2 mm/s, shear distributions at four different time points in the regular (c) and 30% randomized (d) structures. [Color figure can be viewed in the online issue, which is available at http://wileyonlinelibrary.com.]

The distributions of local shear stress throughout the regular and randomized implants are shown in Figure 5 as fluid volume normalized histograms. Shear stress values corresponding to all the interfacial cells are counted and normalized by the total fluid volume. Five key parameters are extracted in Table 2: the mean, standard deviation of shear stress and the mode, skew and kurtosis of the distribution at both early and final growth stages. The strong influence of inflow velocity and strut structure on local shear stress is clearly shown. In the regular structure, more local cells have higher shear stress with the increase of inlet velocity. This trend is also shown for the randomized structure. However, in the 30% randomized structure, both the shifts in modal values of the shear stress from 2.6 to 71.1 mPa at early stage and 5.8 to 71.1 mPa at final stage reveal greater ranges than those in the regular structure [(Figure 5(b)].

**TABLE II:** List of Key Parameters Obtained from Shear Stress Distribution at Final Growth Stage

	Inflow Velocity (mm/s)	Regular		Randomized	
		16.7 h	120 h	16.7 h	120 h
Mean shear stress (mPa)	0.02	4.3	6.7	3.9	6.4
	0.05	13.1	18.5	12.2	19.0
	0.1	30.3	50.1	29.3	48.0
	0.2	66.9	105.2	66.9	90.4
Mode shear stress (mPa)	0.02	5.3	10.2	2.6	5.8
	0.05	11.3	24.5	12.3	12.3
	0.1	35.3	35.3	20.2	54.9
	0.2	60.6	60.6	71.1	71.1
Standard deviation (×10^−3^)	0.02	4.6	6.9	4.8	7.6
	0.05	14.6	20.9	15.9	28.3
	0.1	34.1	86.6	39.3	85.1
	0.2	75.7	304.1	90.9	162.3
Skew	0.02	0.3	0.5	0.7	0.8
	0.05	0.5	0.5	0.8	1.0
	0.1	0.8	1.2	0.9	1.3
	0.2	0.8	1.4	0.9	1.3
Kurtosis	0.02	−1.5	−0.6	−1.1	−0.9
	0.05	−1.4	−0.8	−0.9	−0.4
	0.1	−0.5	1.4	−0.7	0.4
	0.2	−0.5	0.9	−0.8	0.6

In the regular structure, at earlier time points up to 16.7 h [Figure 5(a)], a factor of 10 increase of the inflow velocity (0.02–0.2 mm/s) causes a 11.4 times increase in the mode of the shear stress (5.3–60.6). Although shear stress distributions at later time points, especially in the regular structure, show a less steep increase in the mode of the shear, there is a large difference in distributions between the regular and randomized structures at higher inflow velocities. For example, in Figure 5(c,d), significant reduction in regions with high shear stress values (>1500 mPa) is found in the randomized structures as time increases. And the randomized structure has a broader distribution of shear with a maximum shear stress 1.7 times larger than the regular structure.

**FIGURE 6:**
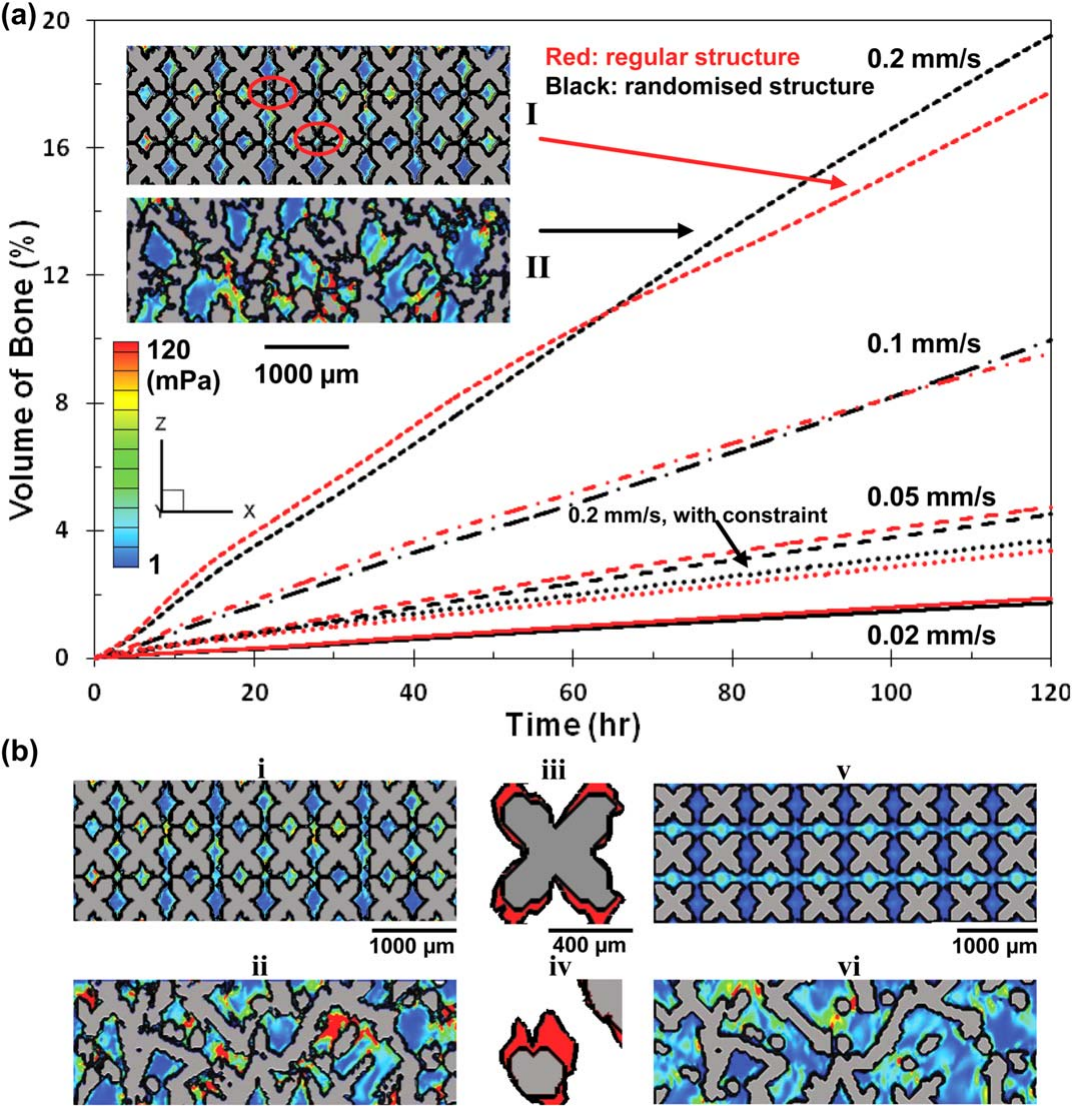
(a) Comparative plot of cellular growth vs. time between the regular and 30% randomized structures at different inflow velocities. Insets: I showing the blockage of the channels in the regular structure at later time stage; II showing the concentration of shear stress in the randomized structure, where indicates more growth at later stages after 70 h. (b) Effect of maximum shear constraint on bone ingrowth at inflow velocity of 0.2 mm/s in the regular and 30% randomized structures. (i,ii) showing the final growth at 120 h without capping the shear stress. (iii,iv) showing zoomed in features of cellular growth (colored red). (v,vi) showing the final growth at 120 h with the shear constraint. Contour colored by shear stress. [Color figure can be viewed in the online issue, which is available at http://wileyonlinelibrary.com.]

A comparative plot of volume fraction occupied by cells over time in the two structures is shown in Figure 6(a). A tenfold increase in inflow velocities causes a nine times increase in cellular growth (1.9–17.5%) in the regular structure after 120 h while in the randomized structure, cellular growth is initially similar for all inflow velocities, but after 60 h, it shows a faster migration with a 11.5 times increase in bone volume (1.7–19.5%).

Several prior experimental studies suggested that there is also a maximum shear stress beyond which cells will not attach and there will be no bone formation. This is simulated by applying the upper shear constraint for cellular growth. Results in Figure 6(b) show significant decreases in cellular growth by 80.1 and 81.0% in the regular and 30% randomized structures with the constraint of maximum shear value, 56 mPa, respectively. Further comparing the two structures, an 8.8% additional decrease of cellular growth volume fraction is found in the randomized structure.

**FIGURE 7:**
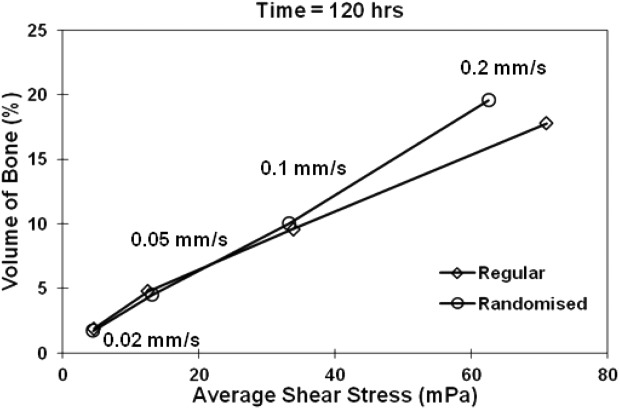
Comparison between the regular and 30% randomized structures at final stage of growth: volume fraction of cellular growth vs. average shear stress at different flow inlet velocities (without shear constraint).

The comparison of the total volume fraction of cellular growth without constraints in both structures is shown in Figure 7 as a function of the average local shear stress. Both regular and 30% randomized structures show similar growth at inflow velocities less than 0.1 mm/s; however by looking at the shear distributions in the 3D structures, factors which may hinder the growth in the regular and randomized structure appear differently, and are discussed in the following section. At an inflow velocity of 0.2 mm/s, after 9% of channel volume is occupied by new grown tissue, the regular structure experiences higher shear stress and shows less increase in bone volume fraction when compared to the randomized structure.

**FIGURE 8:**
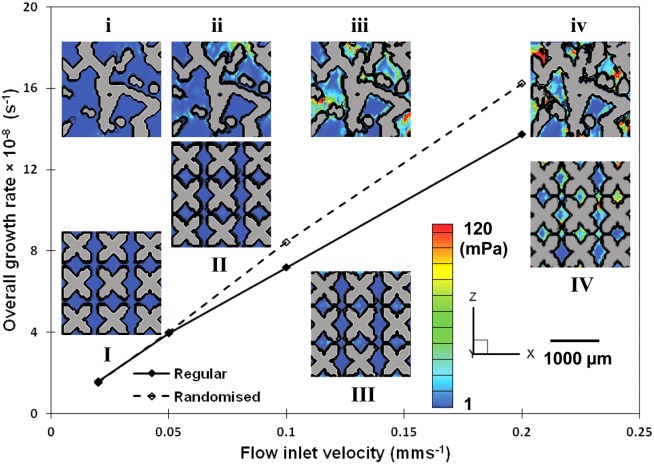
Comparison between the regular and 30% randomized structures: overall growth rate (average value at different time points) versus inflow velocity (without maximum shear constraint). Inset: I, II, III, and IV showing final growth in the regular structure; i, ii, iii, and iv showing the final growth in the randomized structure. Contour colored by shear stress. [Color figure can be viewed in the online issue, which is available at http://wileyonlinelibrary.com.]

The comparison of overall growth rate (calculated by averaging the growth rates at all the time points) for regular and randomized structures is shown in Figure 8. It suggests that at velocities greater than 0.5 mm/s, randomized structure exhibits a generally better performance of cellular growth under the prevalence of the fluid flow induced shear stress.

## DISCUSSION

A numerical model is presented which simulates the microscopic flow shear stress and cell migration, attachment and proliferation (termed *cellular growth* in this paper for brevity), reveals for the first time their complex inter relationship as osteogenesis occurs, especially at a microscopic level. Although a few prior computational approaches have been proposed to evaluate the shear stress on porous scaffold surfaces,^16^,^22^,^29^,^45^ and after imposed bone deposition,^23^ the time dependent changes in flows and distribution of cellular growth have not, to our knowledge, been previously simulated. This model is applied to study complex 3D AM structures, allowing quantitative prediction of the effect of shear stress distribution within the real implant structure on cellular growth, and locates the actual areas where the cellular response takes place.

The model was applied to investigate the local shear stress distribution within two types of implant structures, regular and 30% randomized with four different fluid inflow velocities over a time period of 5 days. As shown in Figure 4(c,d), the randomized structure had a much broader range of shear stress (maximum shear is >4 times that of the regular structure). The distribution of the shear stress in the regular structure showed a regular pattern with relatively high local stress concentration in strut channels, but little shear in areas within pores. This promotes cellular growth in the narrow channels, but not across the bulk of the regular structure. In the randomized structure, the fluid flow was more evenly distributed [Figure 4(b)].

Analysis of shear stress histograms at various time points reveals that in the regular structure, the distribution of shear varied more significantly than that in the randomized structure, especially at later stages of growth [see Figure 5(c,d)]. At lower inflow velocities (0.02 and 0.05 mm/s), there was a twofold increase in the mode of the shear stress with increasing time during the early stages. At higher inflow velocities (0.1 and 0.2 mm/s), a threefold increase in the mode of the shear stress took place in the middle stages. However, after about 9% cellular matrix deposition, the growth rate (mode) dropped and the distribution narrowed. As cellular growth continues, high stress regions were formed (regions with shear greater than 1000 mPa). This indicates that in the regular structure, as the attached cellular layer thickness increases, interconnect regions become constricted, which further reduces shear in the bulk regions (pores).

In the randomized structure, at all inflow velocity magnitudes, the shear stress distributions showed a similar skew trend at all time points. We hypothesize that in the randomized structure preferential flow channels are present (larger channels), which act like larger arteries feeding finer capillaries, where these larger channels do not become as easily constricted as in the regular structure. This results in the magnitude of shear stress increasing during cellular growth without fully obstructing blockages. This shows how internal structural differences may be used as a tool for the bespoke design of implants. For example, at earlier stages of implantation, the structural flow channels need to be formed to serve as a 3D template for guiding the desired cellular growth, and for vascularization. More uniformly distributed shear with a stable average magnitude may be required during later treatment period.

The total amount of cellular growth in both types of structures is compared at different inflow velocities (Figure 6). In general, the amount of cellular growth is directly proportional to the inflow velocity. In the regular structure, the growth increased almost threefold from 1.8 to 4.7%, at inflow speed from 0.02 to 0.05 mm/s. For the randomized design, the result showed similar volume fraction of cellular growth as the regular structure for inflow velocities < 0.1 mm/s. However, at high flow velocities and longer times, the rate of growth in random structures increased by 11% over the regular strut architecture. This finding can be explained by two mechanisms: (1) in the regular structure, due to the ordered design, there are channels where there is little flow in contact with the struts wall and therefore less shear, resulting less amount of cellular matrix in those areas; and (2) as discussed previously, at later growth stages, in the regular design all channels are equally constricted hindering growth, whilst the randomized structure has preferential channels for flow that prevent flow blockages.

Raimondi et al.^16^ suggested that beyond a certain shear stress magnitude, cells would be washed out hence the growth might be hindered. In order to examine the potential of excessive shear stress on bone growth, our numerical model with maximum shear constraint provides a quantitative result on the effect of its cell wash out effect. We picked the simulation with the highest inflow velocity to examine the impact of the maximum shear on cellular growth. It was found that with the maximum shear stress constraint, the amount of cellular matrix decreased significantly in both designs (by more than a factor of 5). This suggests that the high inflow velocity (2 mm/s) simulation results might be unrealistic, and highlights the need for careful selection of profusion bioreactor flow rates. However, the full impact of maximum shear on cellular growth is still unclear, and further experiments need be performed to understand the relationships, that can be used to find the best perfusion inlet velocity and correlate this finding to the cellular growth rate.

The relationship between the volume fraction of cellular growth and the shear stress is shown in Figure 7. Local shear stress increases with the amount of new cellular matrix into the structure, which in turn blocks part of the flow channel, causing fluid to pass through narrower space, resulting in the increase in the fluid velocity in the porous structure and consequently the shear applied on surface cells. It is interesting to see that for the highest inflow velocity case, the regular structure shows a higher local shear profile than the randomized structures for less cellular matrix. This agrees with the observation that at high velocity, regular channels become blocked by fast matrix production, which results in a less optimal environment for later stage cellular growth.

From Figure 8 where we plot the average values of growth rate at different time points with different inflow velocities, it can be concluded that the overall growth rate depends, to a large extent, on the flow inlet velocity. Comparing the performance of the two types of structure, greater overall growth rate may occur at higher flow velocities in a randomized structure. However, the shear stress and the location of the cells and matrix depend greatly on the internal structure at a micro level. Further simulations are required on a statistical basis to test the model on structures with different internal strut and pore morphology.

The model was validated against both prior computational and experimental work. Firstly, the predicted velocity profile was compared to a prior computational fluid dynamics (CFD) simulation given in Ref. (44). The results correlate well, matching the prior study to within 2–16% in terms of the flow velocities and to within 11% in terms of permeability. The models were also compared qualitatively to the studies by Raimondi et al.^17^ and Liu et al.^21^. The current numerical model employs the same concept of time dependent cellular growth kinetics proposed by Liu et al. and Raimondi et al. with the advance of having microscopic shear stress interacting with the cellular growth. Direct comparison of the shear stress magnitudes with the literature is not straightforward since the shear stress estimation will be greatly affected by the choice of simulation parameters and the porosity/microstructure of the structure being tested. Our predicted levels of shear stress agree reasonably well with Raimondi et al.'s work (mean shear stress of 16 mPa at an inlet velocity of 0.22 mm/s).^16^ Compared to Maes et al.'s work^28^ at similar inflow velocity (0.03 mm/s), the average stresses were the same order of magnitude (1.4 mPa). The amount of cellular matrix at 5 days agrees with Raimondi et al.'s prediction but a direct comparison is not possible as the void fraction and shear stress levels were not given in that publication. Note that although we simulated in flow velocities up to 0.2 mm/s to compare, at velocity above 0.1 mm/s the shear stress predicted will be too high for cell attachment.^16^

Although we have used the previously proposed linear cell growth function depending on shear stress, this function can be easily altered in the model as more data becomes available, such as dependency on local oxygen delivery, nutrient concentration and mechanical shear. In addition, the coefficients will be dependent on the fluid, cells used, etc. Another limitation is a paucity of quantitative experimental validation data for these dependencies. At present, we can only compare the average values of shear stress provided by macroscopic computational modeling results. Further modification should be made to our microscopic model to include the nutrition and oxygen transport in order to provide more accurate results of cellular growth with local shear stress distribution once this data becomes available. Improvements such as a statistical analysis can be performed to further test the accuracy of the model by applying the model on different samples with same level of randomization and also samples with different levels of randomization, or only completely different structures, such as apatite foams^46^ or bioglass foams.^47^

In summary, our study provides a microscopic evaluation of shear stress within the implant structure, and reveals direct quantitative results of cellular growth related to shear stress distribution. By simulating the flow in implants with different internal structures, the model provides potential guidelines to optimize the implant construction for stimulating cellular growth.

## CONCLUSIONS

A microscale numerical model, based on the Navier–Stokes equations, was developed to study time dependent osteogenic cell migration, attachment and proliferation (termed *cellular growth* for brevity) and flow shear stress under two proposed AM Ti scaffold structures. The cellular growth as a function of time and shear was simulated in 3D structures on a microscopic scale for the first time and its subsequent influence on the flow was determined.

The influence of local fluid shear stress on cellular growth as a function of increasing inflow velocities, implant structures and time was investigated. The increasing inflow velocity enlarges the range of shear stress and has a positive relationship to the overall growth rate. The results indicate that the 30% randomized structure produces a much higher variation in flow shear than the regular structure. During initial stages of growth, this may not affect osteogenesis significantly; but interestingly as cellular growth progresses, the randomized structure sustains high growth rates due to preferential flow channels forming. In the regular structure, localized growth may hinder the cellular growth at later stage which reduces the overall growth rate. Quantitative effect of excessive shear stress predicted that higher inflow velocities, greater than those shown in experiments, can be used as guidelines for designing the optimal perfusion rates in the cell culturing study. Our model provided a viable tool that can be used to determine the influence of hierarchical structure design in any open cell implant, using additive manufactured Ti implants as an example.

## References

[1] Engh C, Bobyn J, Glassman A (1987). Porous-coated hip replacement. The factors governing bone ingrowth, stress shielding, and clinical results. J Bone Joint Surg Br.

[2] Wolff J, Maquet P, Furlong R, Maquet P, Furlong R (1986). The law of bone remodelling.

[3] Dillaman RM, Roer RD, Gay DM (1991). Fluid movement in bone: Theoretical and empirical. J Biomech.

[4] Reich KM, Frangos JA (1991). Effect of flow on prostaglandin E2 and inositol trisphosphate levels in osteoblasts. Am J Physiol Cell Physiol.

[5] You L, Cowin SC, Schaffler MB, Weinbaum S (2001). A model for strain amplification in the actin cytoskeleton of osteocytes due to fluid drag on pericellular matrix. J Biomech.

[6] Boccaccini AR, Kneser U, Arkudas A (2012). Scaffolds for vascularized bone regeneration: Advances and challenges. Expert Rev Med Devices.

[7] Wray JB, Lynch CJ (1959). The vascular response to fracture of the tibia in the rat. J Bone Joint Surg (Am).

[8] Johnson DL, McAllister TN, Frangos JA (1996). Fluid flow stimulates rapid and continuous release of nitric oxide in osteoblasts. Am J Physiol Endoc.

[9] Weinbaum S, Cowin S, Zeng Y (1994). A model for the excitation of osteocytes by mechanical loading-induced bone fluid shear stresses. J Biomech.

[10] Knothe Tate ML, Knothe, Niederer U (1998). Experimental elucidation of mechanical load-induced fluid flow and its potential role in bone metabolism and functional adaptation. Am J Med Sci.

[11] Sikavitsas VI, Bancroft GN, Holtorf HL, Jansen JA, Mikos AG (2003). Mineralized matrix deposition by marrow stromal osteoblasts in 3D perfusion culture increases with increasing fluid shear forces. Proc Natl Acad Sci USA.

[12] Kapur S, Baylink DJ, William Lau KH (2003). Fluid flow shear stress stimulates human osteoblast proliferation and differentiation through multiple interacting and competing signal transduction pathways. Bone.

[13] Raimondi M, Boschetti F, Falcone L, Fiore G, Remuzzi A, Marinoni E, Marazzi M, Pietrabissa R (2002). Mechanobiology of engineered cartilage cultured under a quantified fluid-dynamic environment. Biomech Model Mech.

[14] Raimondi MT, Boschetti F, Falcone L, Migliavacca F, Remuzzi A, Dubini G (2004). The effect of media perfusion on three-dimensional cultures of human chondrocytes: Integration of experimental and computational approaches. Biorheology.

[15] Cartmell SH, Porter BD, García AJ, Guldberg RE (2003). Effects of medium perfusion rate on cell-seeded three-dimensional bone constructs in vitro. Tissue Eng.

[16] Raimondi MT, Moretti M, Cioffi M, Giordano C, Boschetti F, Laganà K, Pietrabissa R (2006). The effect of hydrodynamic shear on 3D engineered chondrocyte systems subject to direct perfusion. Biorheology.

[17] Raimondi M, Causin P, Mara A, Nava M, Laganà M, Sacco R (2011). Breakthroughs in computational modeling of cartilage regeneration in perfused bioreactors. Biomed Eng.

[18] Cioffi M, Boschetti F, Raimondi MT, Dubini G (2006). Modeling evaluation of the fluid-dynamic microenvironment in tissue-engineered constructs: A micro-CT based model. Biotechnol Bioeng.

[19] Porter B, Zauel R, Stockman H, Guldberg R, Fyhrie D (2005). 3-D computational modeling of media flow through scaffolds in a perfusion bioreactor. J Biomech.

[20] Coletti F, Macchietto S, Elvassore N (2006). Mathematical modeling of three-dimensional cell cultures in perfusion bioreactors. Ind Eng Chem Res.

[21] Liu D, Chua CK, Leong KF (2012). A mathematical model for fluid shear-sensitive 3D tissue construct development. Biomech Model Mech.

[22] Chung C, Chen C, Chen C (2007). Tseng, Enhancement of cell growth in tissue-engineering constructs under direct perfusion: Modeling and simulation. Biotechnol Bioeng.

[23] Lesman A, Blinder Y, Levenberg S (2010). Modeling of flow-induced shear stress applied on 3D cellular scaffolds: Implications for vascular tissue engineering. Biotechnol Bioeng.

[24] Botchwey EA, Pollack SR, El-Amin S, Levine EM, Tuan RS, Laurencin CT (2003). Human osteoblast-like cells in three-dimensional culture with fluid flow. Biorheology.

[25] Jones JR, Poologasundarampillai G, Atwood RC, Bernard D, Lee PD (2007). Non-destructive quantitative 3D analysis for the optimisation of tissue scaffolds. Biomaterials.

[26] Singh R, Lee P, Lindley T, Kohlhauser C, Hellmich C, Bram M, Imwinkelried T, Dashwood R (2010). Characterization of the deformation behavior of intermediate porosity interconnected Ti foams using micro-computed tomography and direct finite element modeling. Acta Biomater.

[27] Raimondi M, Boschetti F, Migliavacca F, Cioffi M, Dubini G, Ashammakhi N, Reis RL (2005). Micro-fluid dynamics in three-dimensional engineered cell systems in bioreactors. Topics Tissue Eng.

[28] Maes F, Van Ransbeeck P, Van Oosterwyck H, Verdonck P (2009). Modeling fluid flow through irregular scaffolds for perfusion bioreactors. Biotechnol Bioeng.

[29] Cioffi M, Küffer J, Ströbel S, Dubini G, Martin I, Wendt D (2008). Computational evaluation of oxygen and shear stress distributions in 3D perfusion culture systems: Macro-scale and micro-structured models. J Biomech.

[30] Yuan L, Lee PD (2010). Dendritic solidification under natural and forced convection in binary alloys: 2D versus 3D simulation. Model Simul Mater Sci.

[31] Wang W, Lee P, McLean M (2003). A model of solidification microstructures in nickel-based superalloys: Predicting primary dendrite spacing selection. Acta Mater.

[32] Lee P, Atwood R, Dashwood R, Nagaumi H (2002). Modeling of porosity formation in direct chill cast aluminum–magnesium alloys. Mat Sci Eng A Struct.

[33] Lee PD, Chirazi A, Atwood R, Wang W (2004). Multiscale modelling of solidification microstructures, including microsegregation and microporosity, in an Al–Si–Cu alloy. Mat Sci Eng A Struct.

[34] Dong H, Lee P (2005). Simulation of the columnar-to-equiaxed transition in directionally solidified Al–Cu alloys. Acta Mater.

[35] Yuan L, Lee PD, Djambazov G, Pericleous K (2009). Numerical simulation of the effect of fluid flow on solute distribution and dendritic morphology. Int J Cast Metal Res.

[36] Yuan L, Lee PD (2012). A new mechanism for freckle initiation based on microstructural level simulation. Acta Mater.

[37] Patankar S (1980). Numerical Heat Transfer and Fluid Flow.

[38] Chorin AJ (1968). Numerical solution of the Navier–Stokes equations. Math Comp.

[39] Al-Rawahi N, Tryggvason G (2002). Numerical simulation of dendritic solidification with convection: Two-dimensional geometry. J Comput Phys.

[40] Granger RA (1995). Fluid Mechanics.

[41] Freed LE, Marquis JC, Langer R, Vunjak-Novakovic G (1994). Kinetics of chondrocyte growth in cell–polymer implants. Biotechnol Bioeng.

[42] Mullen L, Stamp RC, Fox P, Jones E, Ngo C, Sutcliffe CJ (2010). Selective laser melting: A unit cell approach for the manufacture of porous, titanium, bone in-growth constructs, suitable for orthopedic applications. II. Randomized structures. J Biomed Mater Res B.

[43] Mullen L, Stamp RC, Brooks WK, Jones E, Sutcliffe CJ (2009). Selective laser melting: A regular unit cell approach for the manufacture of porous, titanium, bone in-growth constructs, suitable for orthopedic applications. J Biomed Mater Res B.

[44] Zhang Z, Jones D, Yue S, Lee PD, Jones JR, Sutcliffe CJ, Jones E (2013). Hierarchical tailoring of strut architecture to control permeability of additive manufactured titanium implants. Mat Sci Eng C.

[45] Maes F, Van Baelen B, Van Ransbeeck P, Moesen M, Van Oosterwyck H, Verdonck P CFD models for wall shear stress estimation; a comparative study of two complete scaffold geometries.

[46] Jones JR, Hench LL (2003). Regeneration of trabecular bone using porous ceramics. Curr Opin Solid State Mater.

[47] Jones JR, Lee PD, Hench LL (2006). Hierarchical porous materials for tissue engineering. Philos Trans Roy Soc A.

[48] Carvalho DCL, Carvalho MM, Cliquet A (2001). Disuse ostoporosis: its relationship to spine cord injuried patient rehabilitation. Acta Orthop Bras.

[49] Yue S (2011). Non-Destructive quantification of tissue scaffolds and augmentation implants using X-ray microtomography (Doctoral dissertation, Imperial College London).

[50] Yue S, Lee PD, Poologasundarampillai G, Jones JR (2011). Evaluation of 3-D bioactive glass scaffolds dissolution in a perfusion flow system with X-ray microtomography. Acta biomater.

